# A freaky artery

**DOI:** 10.1007/s12471-018-1189-y

**Published:** 2018-10-22

**Authors:** R. Joustra, A. P. J. van Dijk, H. W. J. Meijburg, M. Boulaksil

**Affiliations:** 1 Department of Cardiology, Radboud University Medical Center, Nijmegen, The Netherlands; 2 Department of Cardiology, Jeroen Bosch Hospital, ’s-Hertogenbosch, The Netherlands

## Answer

**Fig. 1a,b:**
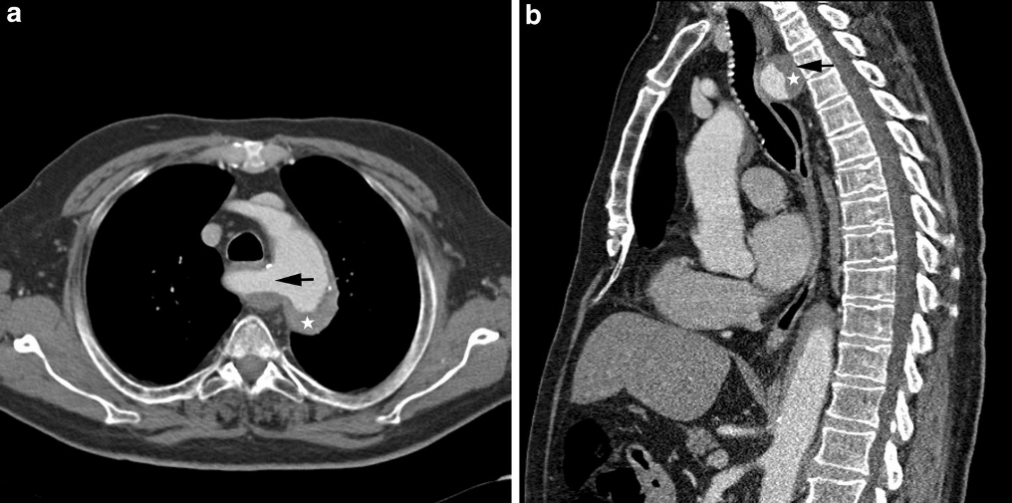
Computed tomography angiography scan performed on admission

The computed tomography angiography (CTA) scan (Fig. 1) demonstrated an intramural haematoma (Fig. 1a, b, asterisk) running from an anomalously originating right subclavian artery (Fig. 1a, b, arrow) continuing to the left aortic arch and descending aorta. The aberrant right subclavian artery, also called *lusorian artery* or *arteria lusoria*, originated at the aortic isthmus beyond the left subclavian artery and passed between the oesophagus and the vertebral column (Fig. 1a, arrow). A 3D volume rendering of the CTA scan is presented as supplementary material.

First described by Hunauld in 1735 [1] and later on associated with dysphagia by Bayford in 1794 [2], the lusorian artery or aberrant right subclavian artery is the most common congenital anomaly of the aortic arch and its thoracic branches with an incidence of 0.4–2% [3], and is considered to be a remnant of the distal portion of the embryonic right aortic arch. When the origin of the lusorian artery is distended, this is called a *Kommerell diverticulum *[4].

Our patient was regarded having a Stanford type B-like aortic dissection and was treated conservatively with antihypertensive medication. During admission, he stayed haemodynamically stable and free of complications and was discharged. Eight months later, a follow-up CTA showed improvement of the intramural haematoma.

Conclusion: intramural haematoma of lusorian artery with ‘Kommerell diverticulum’.

## Caption Electronic Supplementary Material

3D volume rendering of computed tomography angiography scan performed on admission (see also Fig. 1)
